# Humoral and T-cell response 12 months after the first BNT162b2 vaccination in solid organ transplant recipients and controls: Kinetics, associated factors, and role of SARS-CoV-2 infection

**DOI:** 10.3389/fimmu.2022.1075423

**Published:** 2023-01-13

**Authors:** Omid Rezahosseini, Sebastian Rask Hamm, Line Dam Heftdal, Laura Pérez-Alós, Dina Leth Møller, Michael Perch, Johannes Roth Madsen, Annemette Hald, Cecilie Bo Hansen, Jose Juan Almagro Armenteros, Mia Marie Pries-Heje, Rasmus Bo Hasselbalch, Kamille Fogh, Ruth Frikke-Schmidt, Linda Maria Hilsted, Erik Sørensen, Sisse Rye Ostrowski, Zitta Barrella Harboe, Kasper Iversen, Henning Bundgaard, Søren Schwartz Sørensen, Allan Rasmussen, Peter Garred, Susanne Dam Nielsen

**Affiliations:** ^1^ Viro-immunology Research Unit, Department of Infectious Diseases, Rigshospitalet, Copenhagen University Hospital, Copenhagen, Denmark; ^2^ Laboratory of Molecular Medicine, Department of Clinical Immunology, Section 7631, Rigshospitalet, Copenhagen University Hospital, Copenhagen, Denmark; ^3^ Department of Cardiology, Rigshospitalet, Copenhagen University Hospital, Copenhagen, Denmark; ^4^ Department of Clinical Medicine, Faculty of Health and Medical Sciences, University of Copenhagen, Copenhagen, Denmark; ^5^ Novo Nordisk Foundation Center for Protein Research, Faculty of Health and Medical Sciences, University of Copenhagen, Copenhagen, Denmark; ^6^ Department of Cardiology, Herlev and Gentofte Hospital, Copenhagen University Hospital, Copenhagen, Denmark; ^7^ Department of Emergency Medicine, Herlev and Gentofte Hospital, Copenhagen University Hospital, Copenhagen, Denmark; ^8^ Department of Clinical Biochemistry, Rigshospitalet, Copenhagen University Hospital, Copenhagen, Denmark; ^9^ Department of Clinical Immunology, Section 2034, Rigshospitalet, Copenhagen University Hospital, Copenhagen, Denmark; ^10^ Department of Pulmonary and Infectious Diseases, Hospital of North Zealand, Copenhagen University Hospital, Hillerød, Denmark; ^11^ Department of Nephrology, Rigshospitalet, Copenhagen University Hospital, Copenhagen, Denmark; ^12^ Department of Surgical Gastroenterology, Rigshospitalet, Copenhagen University Hospital, Copenhagen, Denmark

**Keywords:** SARS-CoV-2, BNT162 vaccine, mRNA vaccine, humoral immune responses, cellular immune response, organ transplantation

## Abstract

**Introduction:**

We investigated humoral and T-cell responses within 12 months after first BNT162b2 vaccine in solid organ transplant (SOT) recipients and controls who had received at least three vaccine doses. Furthermore, we compared the immune response in participants with and without previous SARS-CoV-2 infection.

**Methods:**

We included adult liver, lung, and kidney transplant recipients, and controls were selected from a parallel cohort of healthcare workers.

**Results:**

At 12th-month, the IgG geometric mean concentrations (GMCs) (P<0.001), IgA GMCs (P=0.003), and median IFN-γ (P<0.001) were lower in SOT recipients than in controls. However, in SOT recipients and controls with previous infection, the neutralizing index was 99%, and the IgG, and IgA responses were comparable. After adjustment, female-sex (aOR: 3.6, P<0.009), kidney (aOR: 7.0, P= 0.008) or lung transplantation (aOR: 7.5, P= 0.014), and use of mycophenolate (aOR: 5.2, P=0.03) were associated with low IgG non response. Age (OR:1.4, P=0.038), time from transplantation to first vaccine (OR: 0.45, P<0.035), and previous SARS-CoV-2 infection (OR: 0.14, P<0.001), were associated with low IgA non response. Diabetes (OR:2.4, P=0.044) was associated with T-cell non response.

**Conclusion:**

In conclusion, humoral and T-cell responses were inferior in SOT recipients without previous SARS-CoV-2 infection but comparable to controls in SOT recipients with previous infection.

## 1 Introduction

Vaccination is an effective and evidence-based strategy to curb the severe acute respiratory coronavirus 2 (SARS-CoV-2) pandemic (1). However, it has been shown that the immune response to SARS-CoV-2 vaccination in immunocompromised persons is lower than in controls (2–5), and solid-organ transplant (SOT) recipients have impaired immune response to SARS-CoV-2 vaccination even after the third or fourth vaccine doses (3, 6–11). Previous studies that investigated the immune response to SARS-CoV-2 vaccination in SOT recipients have focused on humoral immune responses, mainly on anti-SARS-CoV-2 IgG antibodies, with longitudinal follow-ups shorter than six months after first or second vaccine doses (12–17). In a previous study on SOT recipients from Denmark, anti-SARS-CoV-2 IgG antibodies were measured nine months after the second vaccine dose (18). However, there is currently no data about the IgG response in SOT recipients with longer follow-ups after first or second vaccine doses.

IgA antibodies are part of the first-line defense against SARS-CoV-2 infection. It has been shown in controls and SOT recipients that lower serum IgA antibody concentrations after mRNA vaccination are associated with a higher risk of breakthrough infections (19, 20). At present, IgA responses in SOT recipients are less well studied, and there is no information about anti-Spike (S) and receptor-binding domain (RBD) IgA antibodies with follow-up longer than six months after first or second mRNA vaccine doses (21–23).

Besides the humoral immune response, the T-cell response is essential in preventing severe SARS-CoV-2 infection (24–26). It has been shown in controls that the T-cell response lasts for some months after BNT162b2 vaccination, and 73% of controls have detectable T-cell response nine months after the second vaccine dose (25, 26). In SOT recipients, the proportion of T-cell responders within the first month after the second vaccine dose is substantially lower than in controls (27). In the study by Yahav et al., the T-cell response to SARS-CoV-2 spike antigen was measured, and an increase >50 pg/ml interferon-γ (IFN-γ) from baseline was considered positive or adequate. Yahav et al. reported that only 13% of SOT recipients have an adequate T-cell response five months after the second vaccine dose (28). Miele et al. used a SARS-CoV-2 ELISpot Assay to measure T-cell response and a third dose was not found to improve the T-cell response (20).

Vaccination before or after natural infection substantially improves the humoral immune response in controls (29–31). However, studies in SOT recipients that investigated the effects of natural infection on antibody responses had conflicting results (20, 32). Miele et al. reported that natural SARS-CoV-2 infections after a third vaccine dose do not boost IgG and IgA responses (20). Chang et al. found that IgG antibodies in vaccinated SOT recipients who had SARS-CoV-2 infection, was higher than those who did not have infection (32). Therefore, further studies are needed to find the impact of SARS-CoV-2 infection on the humoral and T-cell responses to vaccination.

We aimed to investigate the humoral and T-cell response within 12 months after the first BNT162b2 vaccine dose in SOT recipients who received at least three BNT162b2 vaccine doses and compare the response to the controls. Furthermore, we aimed to compare the immune response in SOT recipients and controls with and without previous SARS-CoV-2 infection. Finally, we aimed to investigate factors associated with humoral and T-cell non response in SOT recipients.

## 2 Materials and methods

### 2.1 Study design

In this prospective observational cohort study, we followed SOT recipients twelve months after the first BNT162b2 vaccine dose. SARS-CoV-2 vaccination in Denmark was initiated on December 27^th^, 2020, and SOT recipients were prioritized for early vaccination. All adult liver, lung, and kidney transplant recipients followed at Copenhagen University Hospital, Rigshospitalet, were invited to participate in this study. From January 2021 through April 2021, the study was open for inclusion for SOT recipients who did not receive the first BNT162b2 vaccine dose or those who received the first dose but not the second dose. From July 2021, all liver, lung, and kidney transplant recipients followed at Copenhagen University Hospital, Rigshospitalet, were invited to participate regardless of vaccination status. Participation in the study was voluntary and did not interfere with the Danish vaccination strategy. Controls were selected from a parallel study of healthcare workers at Rigshospitalet and Herlev-Gentofte Hospital, as previously described (33, 34). We excluded participants who were vaccinated with less than three doses and those who were vaccinated with vaccines other than the BNT162b2. Furthermore, due to the risk of cross reactivity between IgG antibodies generated after vaccination and the IgG antibodies administrated for SARS-CoV-2 treatment, participants who received monoclonal antibody treatment were excluded from IgG analyses, but not from IgA or T-cell analyses. It has been suggested that monoclonal antibodies clear or block antigens, hide antigen-epitopes from immune cells, and result in a hindered endogenous IgG response but not the T cell response (35–37).

The study was performed in accordance with the declaration of Helsinki. All participants provided written and oral informed consent, and the institutional review board approved the study at the Regional Scientific Ethics Committee of the Capital Region of Denmark (H-20079890).

### 2.2 Clinical information

Information on demographics, transplantation-related variables, medication, comorbidities, and treatment for acute rejections was collected from medical records. Information on vaccinations was collected from the Danish Vaccination Register (DDV) (38). Since 2015, it has been mandatory to register all vaccinations administered in Denmark in DDV. Data on SARS-CoV-2 RT-PCR results were collected from the Danish Microbiology Database (MiBa) (39), which has complete national coverage, including information from samples collected in the primary sector, hospitals, and SARS-CoV-2 test centers.

### 2.3 Blood sampling

Blood samples were part of the Vaccination Clinic for Immunocompromised (VACCIM) project, following anti-SARS-CoV-2 vaccine responses in patients and health care personnel from the Capital Region of Denmark, which were collected at baseline, three weeks, two months, six months, and twelve months after administrating the first BNT162b2 vaccine dose. The second BNT162b2 vaccine dose was injected at least 14 days after the first dose. Therefore, baseline samples were collected before or up to 13 days after the first vaccine dose. Furthermore, to include as many participants as possible, the 3^rd^-week, 2^nd^-month, 6^th^-month, and 12^th^-month samples were collected from days 14 to 33, 34 to 90, 91 to 273, and 274 to 456 after administrating the first BNT162b2 vaccine dose, respectively.

We previously described and reported data on anti-RBD IgG kinetics from the first six months of follow-up and T-cell responses from the six-month follow-up point only (34).

### 2.4 Determination of antibodies

The anti-RBD IgG and anti-RBD IgA concentrations in venous blood were measured using an in-house direct ELISA as previously described (33, 34, 40). The RBD of the SARS-CoV-2 Spike protein plays a crucial role in the cell entry-mechanism necessary for viral replication (41). This means that the RBD of the SARS-CoV-2 Spike protein is an important functional target of anti-SARS-CoV-2 antibodies (42). By measuring antibodies binding to the SARS-CoV-2 Spike protein RBD we measure antibodies with potential to neutralize SARS-CoV-2 virus replication (42, 43). Furthermore, an in-house pseudo neutralization ELISA was used to estimate the neutralizing capacity of antibodies against the ancestral (Wuhan) strain of SARS-CoV-2 as previously described (33, 34, 43). Specific antibodies against the SARS-CoV-2 nucleocapsid (N) antigen not included in the vaccines used in Denmark were measured using the Elecsys^®^ Anti-SARS-CoV-2 immunoassay (Roche Diagnostics GmbH, Germany) and a Cobas 8000 analyzer system (Roche Diagnostics), according to the manufacturer’s instructions.

### 2.5 Determination of T-cell response by interferon gamma releasing assay

To determine the T-cell response, T-cells from fresh whole blood were stimulated with SARS-CoV-2 spike protein 1 (S1) using a commercial kit (ET 2606-3003, EUROIMMUN, Lübeck, Germany). Then, we measured interferon-γ (IFN-γ) release after stimulation using a commercial kit (EQ 6841-9601, EUROIMMUN, Lübeck, Germany), according to manufacturer’s instructions and as described previously (33, 34).

### 2.6 Definitions

A positive anti-RBD IgG response was defined as more than 25% inhibition in the pseudo-neutralizing assay and concurrent anti-RBD IgG concentration above 225 arbitrary units per milliliter (AU/mL) (44). A positive anti-RBD IgA response was defined as having an anti-RBD IgA concentration above 100 AU/mL. These cutoffs were determined based on a receiver operating characteristic curve (ROC) as was described by Hansen et al. (40). A positive T-cell response was defined as an IFN-γ concentration above 200 milli-international units per milliliter (mIU/mL), according to the manufacturer’s instructions.

Sampling time was defined as the time from the first BNT162b2 vaccine dose to the blood sample collection.

SARS-CoV-2 infection within 12 months was defined as either the presence of nucleocapsid (N)-antibodies in the 12^th^-month sample and/or a positive SARS-CoV-2 RT-PCR prior to the 12^th^-month sample.

Monoclonal antibody treatment was defined as treatment with sotrovimab (GlaxoSmithKline pharmaceutical and biotechnology company, London, England) or REGN-COV2 (Roche pharmaceutical company, Basel, Switzerland) within 90 days of the 12^th^-month sample.

To apply similar and comparable criteria for all SOT recipients, we considered the immunosuppressive regimen at the first BNT162b2 vaccination as the maintenance immunosuppressive therapy.

Fourteen days after the third, or the fourth dose of BNT162b2, a participant was considered vaccinated with three, or four vaccine doses, respectively.

### 2.7 Statistics

Continuous data were reported as medians with interquartile range (IQR), and categorical data were reported as numbers and proportions. The normality of data distribution was assessed using quantile-quantile plots.

To compare anti-RBD IgA geometric mean concentrations (GMCs), we matched 204 SOT recipients with 204 controls on age and sex (**Supplementary Tables**).

We then fitted a two-part linear mixed model with log-transformed anti-RBD IgA concentration as the dependent variable and sampling time, SOT recipient or control status, and previous SARS-CoV-2 infection (yes/no) as fixed effects allowing for interaction between these variables. SOT recipients and controls were divided into those with- or without previous SARS-CoV-2 infection from the first sample with evidence of infection (positive N-antibodies or SARS-CoV-2 RT-PCR). The two-part mixed model was composed of a zero-inflation model, which models the probability of an observation being zero, and a conditional model, which models the anti-RBD IgA concentration for non-zero observations (45). The model and the residuals were checked using the DHARMa package.

Twenty-three out of 204 SOT recipients received monoclonal antibodies and therefore were excluded from comparisons for anti-RBD IgG analyses. To compare the anti-RBD IgG GMCs between SOT recipients and controls, matching was done on 181 SOT recipients and 181 controls, and then the two-part linear mixed model was run as described above (**Supplementary Tables**).

It was impossible to extract p-values from the two-part linear mixed model and statistically compare the groups of infected/non-infected participants. Therefore, we compared the observed anti-RBD IgG and anti-RBD IgA antibody GMCs between infected/non-infected groups at 12^th^-month using the Mann-Whitney U test. In sensitivity analyses modeling of anti-RBD IgG and anti-RBD IgA GMCs and Mann-Whitney U test were repeated after excluding SOT recipients who received four doses of vaccine.

Due to the missing data for baseline, 3^rd^-week, and 2^nd^-month after the first vaccine dose, running a mixed model for IFN-γ concentration was impossible. IFN-γ concentration at 12^th^-month was available for 169 SOT recipients and was compared to 169 age- and sex-matched controls using the Mann-Whitney U test (**Supplementary Tables**).

We used uni- and multivariable logistic regression models to investigate variables associated with anti-RBD IgG, anti-RBD IgA, or IFN-γ non responses in SOT recipients. The multivariable models were adjusted for sex, transplant type, time from transplantation to vaccination, number of vaccine doses, prior SARS-CoV-2 infection, antimetabolite treatment, corticosteroid treatment, and diabetes mellitus.

A p-value <0.05 was considered significant. Analyses were performed using R version 4.0.3 (R Core Team, 2020, Vienna, Austria) and package glmmTMB (45).

## 3 Results

### 3.1 Characteristics of SOT recipients

We included 204 SOT recipients; of those, 93 (46%) were kidney, 74 (36%) liver, and 37 (18%) lung transplant recipients. Characteristics of SOT recipients are presented in **Table 1**. The median age at the first BNT162b2 vaccine dose was 59 years (IQR 55-64), and 118 (58%) were male. The median (IQR) time from transplantation to the first vaccine dose was 6.3 years (2.8-11) (**Table 1**).

**Table 1:** Patients characteristics.

		Total (n=204)	Kidney transplant recipients (n=93)	Lung transplant recipients (n=37)	Liver transplant recipients (n=74)
**Age (years), median (IQR**		59 (55-64)	57 (52-64)	59 (55-64)	58 (51-64)
**Sex (male), n (%)**		118 (58)	56 (60)	17 (46)	45 (61)
**Time from transplantation to first vaccine dose, median (IQR)**		6.3 (2.8-11)	7.7 (3.0-12)	4.1 (1.8-7.9)	6.2 (3.3-11)
**Comorbidities (n) (%)**	Cardiovascular disease ^†^	138 (68)	81 (87)	27 (73)	30 (41)
	Pulmonary disease	22 (11)	7 (7.5)	5 (14)	10 (14)
	Diabetes mellitus	42 (21)	20 (22)	5 (14)	17 (23)
**Immunosuppressive treatment, n (%)**	No antimetabolites	33 (16)	11 (12)	10 (27)	12 (16)
	Mycophenolate	147 (72)	68 (73)	22 (60)	57 (77)
	Azathioprine	24 (12)	14 (15)	5 (14)	5 (6.8)
	Calcineurin inhibitor (Ciclosporin, Tacrolimus)	168 (82)	80 (86)	25 (68)	63 (85)
	mTOR inhibitor (Sirolimus, Everolimus)	21 (10)	11 (12)	0 (0.0)	10 (14)
	CNI and mTOR inhibitor ^†^	12 (5.9)	1 (1.1)	11 (30)	0 (0.0)
	Corticosteroids ^†^	153 (75)	85 (91)	33 (89)	35 (47)
**Vaccine doses prior to 12-month sample, n (%)**	Three	173 (85)	80 (86)	34 (92)	59 (80)
	Four	31 (15)	13 (14)	3 (8.1)	15 (20)
**Time from last vaccine dose, days, median (IQR)**		127 (113-137)	126 (115-137)	130 (123-137)	123 (82-137)
**High-dose methyl-prednisone ^‡^**		5 (2.5)	2 (2.2)	3 (8.1)	0 (0.0)
**SARS-CoV-2 infection prior to 12-months sample, n (%)**		49 (24)	22 (24)	8 (22)	19 (26)

^†^ P<0.001.

^‡^ High-dose methyl-prednisone was defined as per protocol high dose steroid for treatment of acute rejection or suspected acute rejection, P=0.029.

Within the 12 months after the first BNT162b2 vaccine dose, 173 (85%) out of 204 SOT recipients received three vaccine doses, and 31 (15%) received four vaccine doses. All 204 controls received three vaccine doses.

Forty-nine (24%) and 59 (29%) out of 204 SOT recipients and 204 controls, respectively, had at least one episode of SARS-CoV-2 infection before the 12^th^-month (P=0.313).

Twenty-three (11%) out of 204 SOT recipients received monoclonal antibodies and were excluded from IgG analyses. One SOT recipient received REGN-COV2, and 22 SOT recipients received Sotrovimab.

### 3.2 Kinetics of anti-RBD IgG antibodies

**Figure 1:**
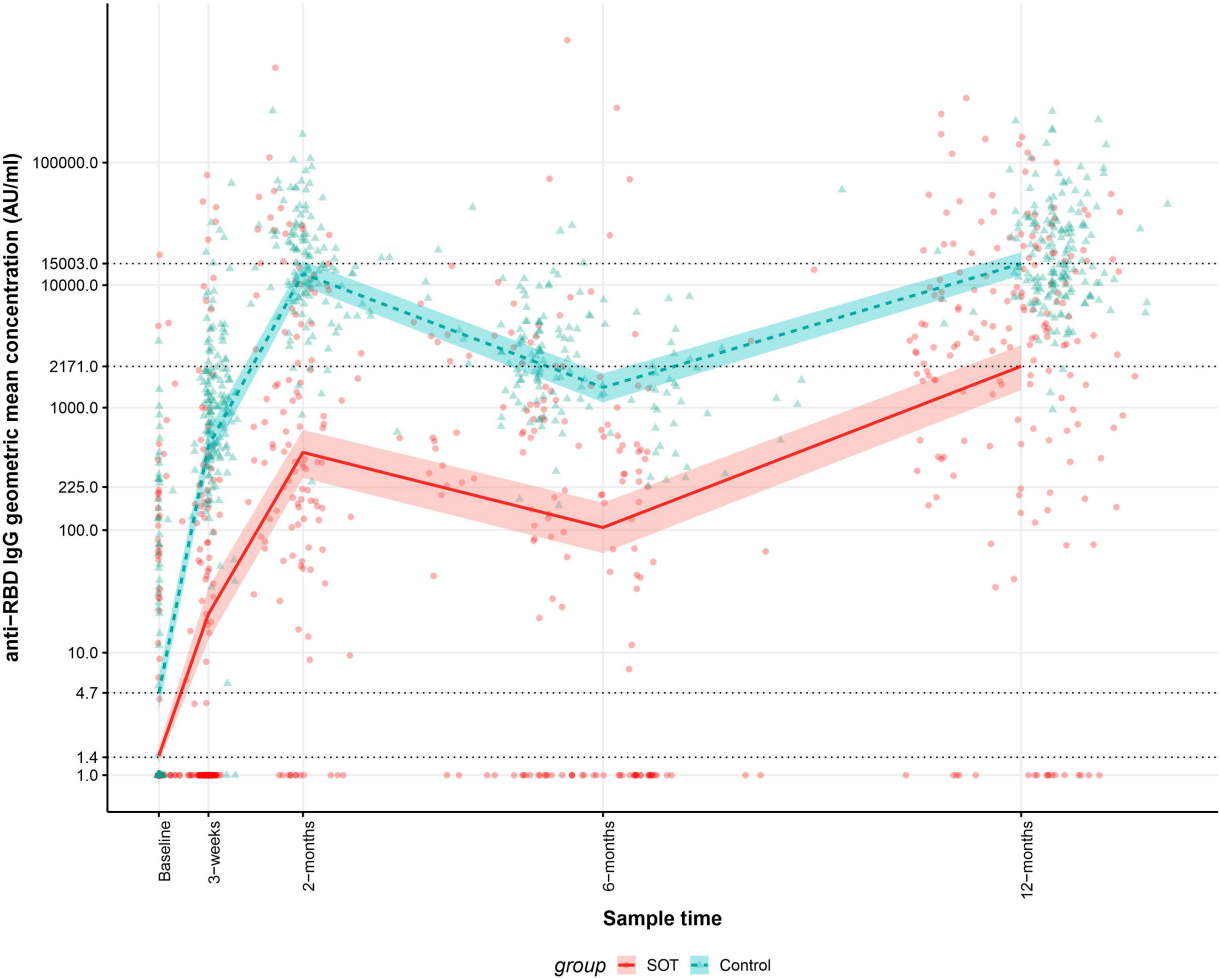
Kinetics of the modeled anti-receptor-binding domain (RBD) Immunoglobulin G (IgG) geometric mean concentrations (GMCs) in solid organ transplant (SOT) recipients and controls within 12 months after the first BNT162b2 dose and observed anti-RBD IgG concentrations. The data include 189 SOT recipients (red) and 189 age and sex matched controls (blue). The modeled anti-RBD IgG GMCs in AU/mL with 95% confidence intervals are plotted on top of each individual sample’s measured anti-RBD concentration in AU/mL. The y-axis is on a log10 scale. The dashed horizontal lines indicate modeled anti-RBD IgG GMC values for SOT recipients and controls at baseline and 12 months after the first BNT162b2 dose”.

In order to determine differences in the anti-RBD IgG GMCs in SOT recipients and controls with and without previous SARS-CoV-2 infections 12 months after the first vaccine dose, the development in anti-RBD IgG GMCs from baseline to 12 months after the first vaccine dose was analyzed. Modeled anti-RBD IgG GMCs were lower in SOT recipients than in controls at all-time points. **Figure 1** shows that the modeled baseline anti-RBD IgG GMCs were 1.4 (95% CI: 1.3-1.7) AU/mL and 4.7 (95% CI: 3.5-6.5) AU/mL for SOT recipients and controls, respectively (p=0.008). At 12^th^-month, the GMCs were 2,171 (95% CI: 1405-3213) AU/mL and 15,003 (12,100–18,511) AU/mL for SOT recipients and controls, respectively (p<0.001).

**Figure 2:**
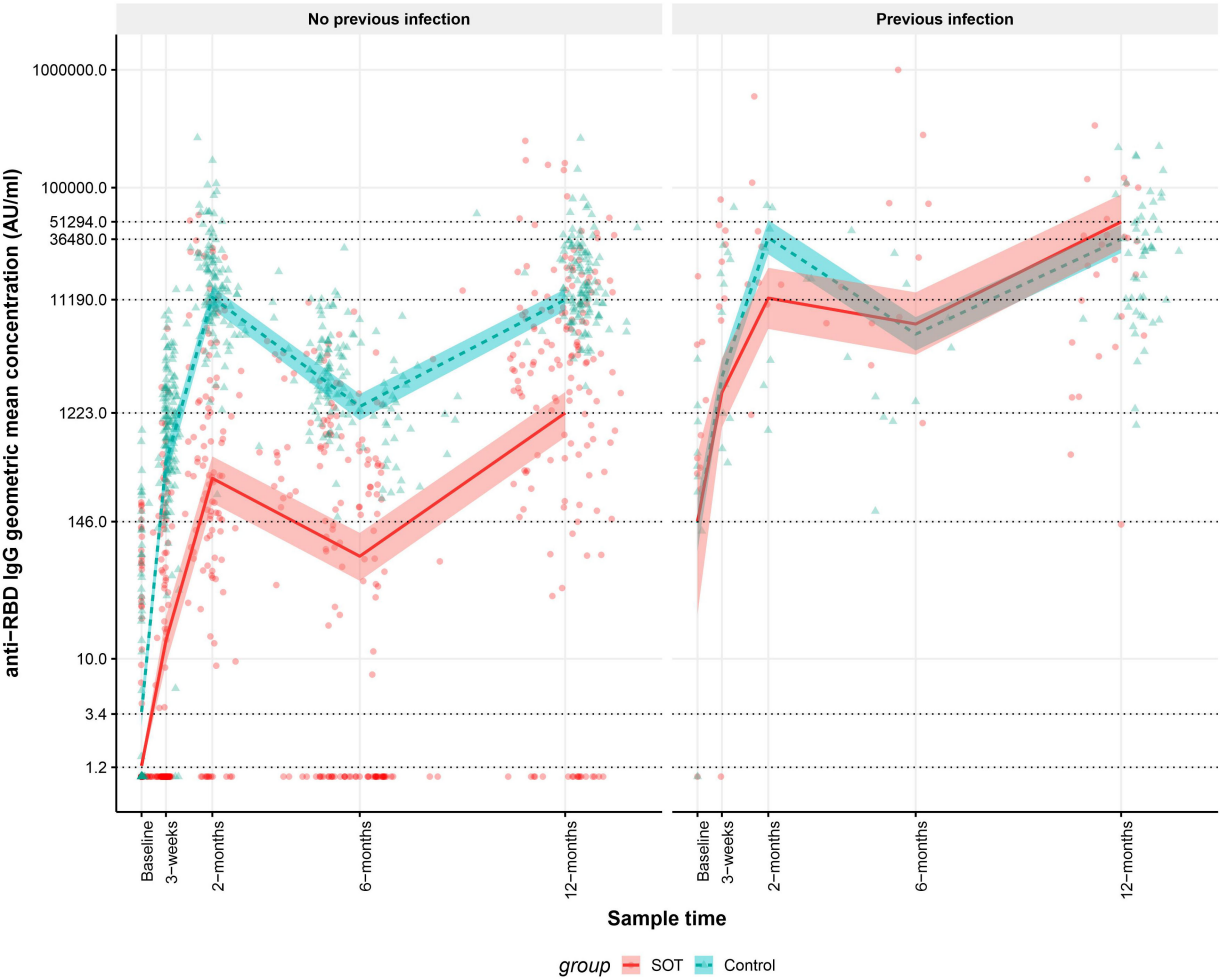
Kinetics of the modeled anti-receptor-binding domain (RBD) Immunoglobulin G (IgG) geometric mean concentrations (GMCs) in solid organ transplant (SOT) recipients and controls with and without previous infection within 12 months after the first BNT162b2 dose and observed anti-RBD IgG concentrations. The data include 189 SOT recipients (red) and 189 age and sex matched controls (blue). A sample is defined as previously infected when an individual has a positive SARS-CoV-2 PCR test prior to the sample or SARS-CoV-2 Nucleocapsid antibodies are present in the sample or a previous sample from the same individual. The modeled anti-RBD IgG GMCs in AU/mL with 95% confidence intervals are plotted on top of each indiviudal sample’s measured anti-RBD concentration in AU/mL. The y-axis is on a log10 scale. The dashed horizontal lines indicate modeled anti-RBD IgG GMC values for SOT recipients and controls with and without prior infection at baseline and 12 months after first BNT162b2 dose.

We compared the modeled anti-RBD IgG GMCs in SOT recipients and controls with and without SARS-CoV-2 infection. In SOT recipients and controls without previous SARS-CoV-2 infection anti-RBD IgG GMCs were lower in SOT recipients than in controls at all-time points. In SOT recipients and controls with previous SARS-CoV-2 infections, anti-RBD IgG GMC were similar at all-time points except from 2 months after the first vaccine dose. As **Figure 2** shows, in SOT recipients and controls with the previous infection, modeled anti-RBD IgG GMCs were 146 (95% CI: 24-555) AU/mL and 142 (95% CI: 82-230) AU/mL (p=0.327) at baseline, while modeled anti-RBD IgG GMCs were 51,294 (95% CI: 30,092-87,428) and 36,480 (95% CI: 27,856-48,164) (p=0.436), respectively, 12 months after first vaccine dose. At baseline SOT recipients and controls without previous SARS-CoV-2 infection had modeled anti-RBD IgG GMCs of 1.2 (95% CI: 1.1-1.4) AU/mL and 3.4 (95% CI: 2.7-4.8) AU/mL, respectively (p=0.038) while modeled anti-RBD IgG GMCs were 1223 (95% CI: 762-1842) and 11,190 (95% CI: 8994-13,791) 12 months after first vaccine dose (p<0.001). No controls received four vaccine doses; therefore, sensitivity analyses excluding SOT recipients who received four doses of vaccine was done. The modeled antibody concentrations remained within the confidence intervals of the reported values.

### 3.3 Kinetics of anti-RBD IgA antibodies

**Figure 3:**
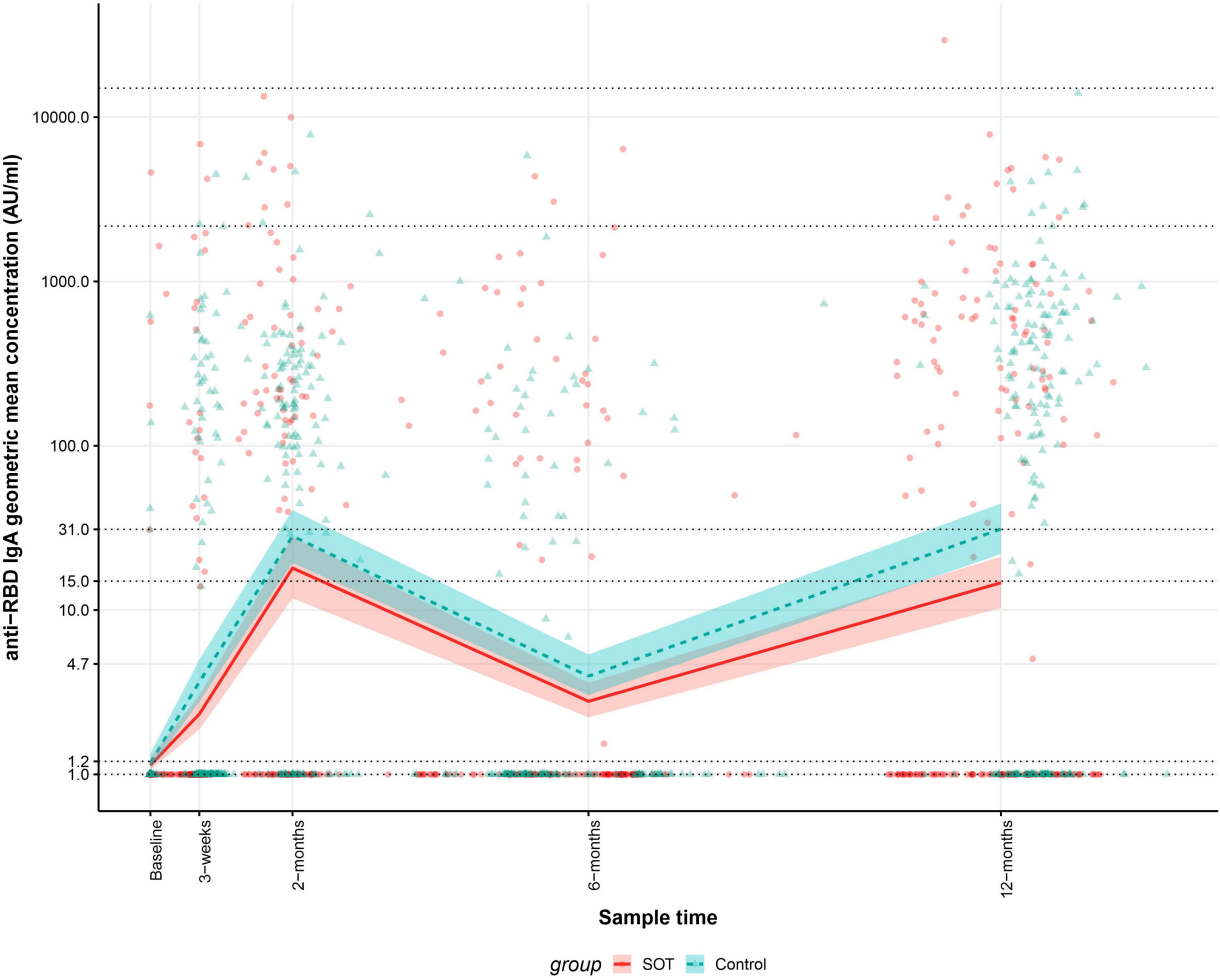
Kinetics of the modeled anti-receptor-binding domain (RBD) Immunoglobulin A (IgA) geometric mean concentrations (GMCs) in solid organ transplant (SOT) recipients and controls within 12 months after the first BNT162b2 dose and observed anti-RBD IgG concentrations. The data include 204 SOT recipients (red) and 204 age and sex matched controls (blue). The modeled anti-RBD IgA GMCs in AU/mL with 95% confidence intervals are plotted on top of each indiviudal sample’s measured anti-RBD concentration in AU/mL. The y-axis is on a log10 scale. Dashed horizontal lines indicate modeled anti-RBD IgA GMC values for SOT recipients and controls at baseline and 12 months after first BNT162b2 dose.

In order to determine differences in the anti-RBD IgA GMCs in SOT recipients and controls with and without previous SARS-CoV-2 infections 12 months after the first vaccine dose, the development in anti-RBD IgA GMCs in from baseline to 12 months after the first vaccine dose was analyzed. Modeled anti-RBD IgA GMC was lower in SOT recipients than in controls 12 months after the first vaccine dose and similar at all other time points. As **Figure 3** shows, The baseline anti-RBD IgA GMCs were 1.1 (95% CI: 1.1-1.3) AU/mL and 1.2 (95% CI: 1.1-1.3) AU/mL for SOT recipients and controls, respectively (p=0.083). At 12^th^-month, the GMCs were 15 (95% CI: 10-21) AU/mL and 31 (22–44) AU/mL for SOT recipients and controls, respectively (p=0.002).

**Figure 4:**
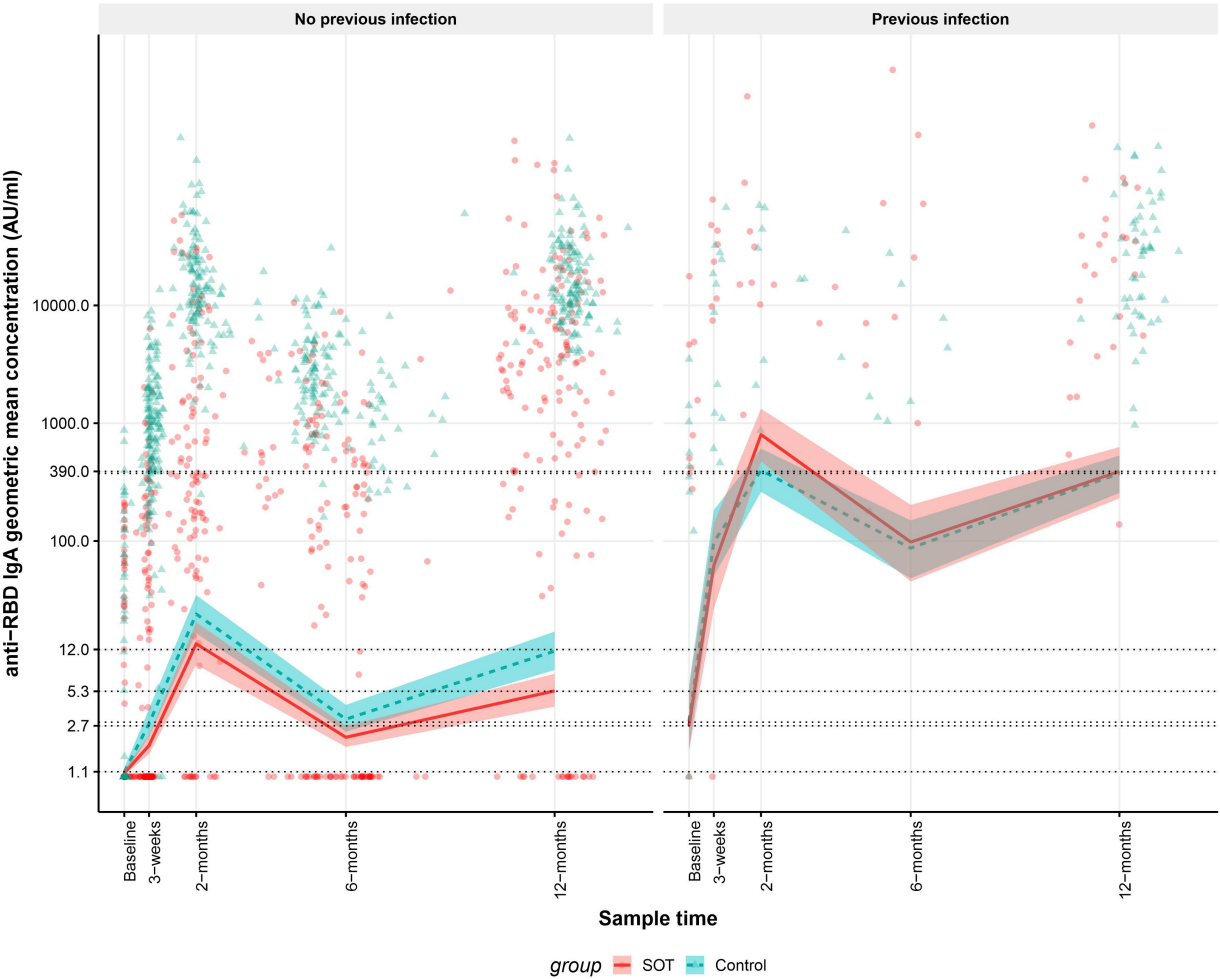
Kinetics of the modeled anti-receptor-binding domain (RBD) Immunoglobulin A (IgA) geometric mean concentrations (GMCs) in solid organ transplant (SOT) recipients and controls with and without previous infection within 12 months after the first BNT162b2 BNT162b2 and observed anti-RBD IgG concentrations. The data include 204 SOT recipients (red) and 204 age and sex matched controls (blue). A sample is defined as previously infected, when an individual has a positive SARS-CoV-2 PCR test prior to the sample or SARS-CoV-2 Nucleocapsid antibodies are present in the sample or a previous sample from the same individual. The modeled anti-RBD IgA GMCs in AU/mL with 95% confidence intervals are plotted on top of each indiviudal sample’s measured anti-RBD concentration in AU/mL. The y-axis is on a log10 scale. The dashed horizontal lines indicate modeled anti-RBD IgA GMC values for SOT recipients and controls with and without prior infection at baseline and 12 months after first BNT162b2 dose.

In SOT recipients and controls without previous SARS-CoV-2 infection anti-RBD IgA GMC was lower in SOT recipients than in controls 12 months after the first vaccine dose and similar at all other time points. In SOT recipients and controls with previous SARS-CoV-2 infections, anti-RBD IgG GMC were similar at all-time points. As **Figure 4** shows, in SOT recipients and controls with previous infection, modeled anti-RBD IgA GMCs were 2.7 (95% CI: 1.6-5.6) AU/mL and 2.9 (95% CI: 1.7-6.0) AU/mL (p=0.377) at baseline while modeled anti-RBD IgA GMCs were 390 (95% CI: 230-628) and 375 (95% CI: 256-532) (p=0.597) 12 months after first vaccine dose. At baseline SOT recipients and controls without previous SARS-CoV-2 infection had modeled anti-RBD IgA GMCs of 1.1 (95% CI: 1.0-1.1) AU/mL and 1.1 (95% CI: 1.0-1.2) AU/mL, respectively (p = 0.170) while modeled anti-RBD IgG GMCs were 5.3 (95% CI: 3.9-7.5) and 12 (95% CI: 8.0-17) 12 months after first vaccine dose (p<0.012). No controls received four vaccine doses; therefore, sensitivity analyses excluding SOT recipients who received four doses of vaccine was done. The modeled antibody concentrations remained within the confidence intervals of the reported values.

### 3.4 Neutralizing index

The median neutralizing index in SOT recipients and controls in the 12^th^-month sample was 98.9% (IQR: 93.0-99.4) and 99.3% (IQR: 98.6-99.6) (p<0.001). In SOT recipients and controls with previous SARS-CoV-2 infection, the median neutralizing index was 99.3% (IQR: 98.6-99.5) and 99.4% (IQR: 98.6-99.6) (p=0.285), respectively. In SOT recipients and controls without previous infection, the median neutralizing index was 98.7% (IQR: 71.9-99.4) and 99.2% (IQR: 98.6-99.6) (p<0.001), respectively.

### 3.5 T-cell response

To determine differences in the T-cell responses between SOT recipients and controls with and without previous SARS-CoV-2 infections, we compared spike-specific IFN- γ concentrations 12 months after the first vaccine dose. Twelve months after the first vaccine dose, 77 (46%) out of 169 SOT recipients and 117 (69%) out of 169 controls had an IFN-γ response higher than the cut-off (p=0.005). The median (IQR) IFN-γ was 110 mIU/mL (IQR 0-831) and 1156 mIU/mL (IQR: 488-2,918) for SOT recipients and controls, respectively (p<0.001). Forty-three of 169 SOT recipients and 40 of 169 controls had SARS-CoV-2 infection prior to the 12^th^ month sample, with median IFN-γ of 775 mIU/mL (IQR 61-3,509) and 2,657 mIU/mL (IQR 758-5,511), respectively (p=0.018).

**Figure 5:**
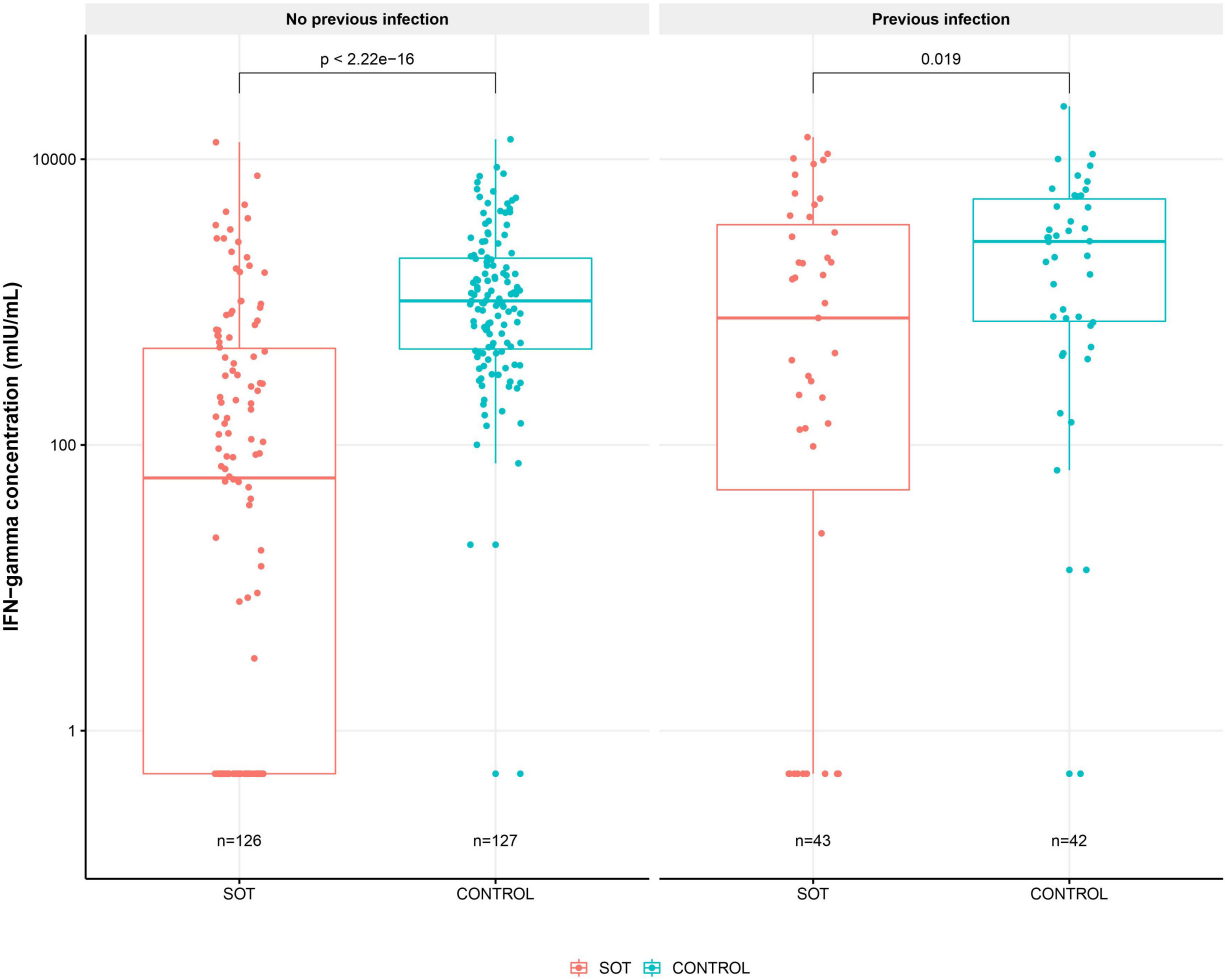
SARS-CoV-2 Spike protein specific T-cell response 12 months after first BNT162b2 dose in solid organ transplant (SOT) recipients and controls with and without previous SARS-CoV-2 infection. Boxplots of spike-specific interferon gamma concentration in mIU/mL in solid organ transplant (SOT) recipients and controls with and without previous infection 12 months after first vaccine dose. The y axis shows spike-specific interferon gamma concentration in mIU/mL on a log10 scale and the x axis the number of SOT recipients and controls in each group. Differences between SOT recipients and controls was tested with Mann-Whitney U test and p values shown on the plot.

In 34 of 169 SOT recipients and 77 of 169 controls without previous infection, median IFN-γ was 59 mIU/mL (IQR: 0-475) and 1,020 mIU/mL (IQR: 456-1,984) (p<0.001). **Figure 5** shows IFN-γ concentrations at 12^th^-month in SOT recipients and controls with and without previous SARS-CoV-2 infection.

### 3.6 Risk factors for anti-RBD IgG non response in SOT recipients

To determine risk factors of anti-RBD IgG non-response 12 months after first vaccine dose, logistic regression analyses were performed. The univariable models are showed in **Table 2**. In the multivariable model, female SOT recipients and SOT recipients with diabetes mellitus had an OR of 3.6 ([95% CI, 1.4-9.5], P=0.009), and 4.8 ([95% CI, 1.6-15], P= 0.006) for anti-RBD IgG non response. Moreover, in comparison with liver transplant recipients, kidney and lung transplant recipients had an OR of 7.0 ([95% CI, 1.7-29], P= 0.001), and 7.5 ([95% CI, 1.5-37], P= 0.014) for anti-RBD IgG non response, respectively. SOT recipients who received mycophenolate had an OR of 5.2 ([95% CI, 1.6-15], P=0.006) for anti-RBD IgG non response (**Table 2**).

**Table 2:** Risk factors for anti-RBD IgG non response.

	Unadjusted model			Adjusted model*		
	OR	95% CI	P-value	OR	95% CI	P-value
**Age per ten years**	1.27	0.90-1.8	0.178	1.7	0.99-2.7	0.053
**Female sex**	**2.6**	**1.2-5.7**	**0.012**	**3.6**	**1.4-9.5**	**0.009**
Transplantation type (Liver Tx as reference)						
Kidney Lung	**8.58** **9.43**	**2.5-30** **2.3-38**	**0.001** **0.002**	**7.0** **7.5**	**1.7-29** **1.5-37**	**0.008** **0.014**
**Time from transplantation to first vaccine dose (per one year increase)**	0.96	0.91-1.02	0.157	0.99	0.92-1.1	0.786
Number of vaccines before the infection (Three doses as reference)						
- Four	0.54	0.18-1.7	0.286	0.84	0.22-3.2	0.797
**Time from last vaccine dose (more than 120 days as reference)**	0.88	0.40-1.9	0.752	–	–	–
**SARS-CoV-2 infection prior to 12^th^ month-sample**	0.14	0.02-1.1	0.060	0.12	0.01-1.04	0.054
Immunosuppressive treatment						
Antimetabolite (None as reference)						
- Mycophenolate - Azathioprine	1.7 1.4	0.56-5.4 0.30-6.2	0.340 0.683	**5.2** 1.3	**1.2-23** 0.23-7.6	**0.030** 0.771
Corticosteroids	**4.8**	**1.4-16**	**0.013**	3.1	0.68-14	0.145
CNI or mTOR (CNI as reference)						
mTOR	0.17	0.02-1.3	0.092	–	–	–
Comorbidities						
**Cardiovascular disease**	1.5	0.66-3.5	0.336	–	–	–
**Diabetes mellitus**	**2.7**	**1.2-6.0**	**0.020**	**4.8**	**1.6-15**	**0.006**

*Adjusted for sex, transplant type, time from transplantation, number of vaccine doses, prior SARS-CoV-2 infection, antimetabolite treatment, corticosteroid treatment, and diabetes mellitus. P-values for the bold values were <0.05 (statistically significant).

### 3.7 Risk factors for anti-RBD IgA non response

To determine risk factors of anti-RBD IgA non-response 12 months after first vaccine dose, logistic regression analyses were performed. The univariable models are showed in **Table 3**. In the multivariable model, older age, increase in time from transplantation to first vaccine dose, and having SARS-CoV-2 infection before the 12^th^-month sample had an OR of 1.4 per ten-year increase ([95% CI, 1.02-1.9], p = 0.038), 0.95 per one year ([95% CI, 0.91-0.99], P=0.035) and 0.14 ([95% CI, 0.07-0.30], P<0.001) for anti-RBD IgA non response, respectively (**Table 3**).

**Table 3:** Risk factors for anti-RBD IgA non response.

	Unadjusted model				Adjusted model**			
	OR		95% CI	P-value	OR		95% CI	P-value
**Age per ten years**	1.2		0.94-1.6	0.145	**1.4**		**1.02-1.9**	**0.038**
**Female sex**	1.4		0.76-2.4	0.312	1.4		0.70-2.7	0.356
Transplantation type (Liver Tx as reference)								
- Kidney - Lung	1.7 **2.4**		0.92-3.2 **1.02-5.7**	0.093 **0.043**	1.5 1.8		0.67-3.3 0.64-5.0	0.324 0.267
**Time from transplantation to first vaccine dose**	**0.95**		**0.92-0.99**	**0.021**	**0.95**		**0.91-0.99**	**0.035**
Number of vaccines before the infection (Three doses as reference)								
- Four		1.01	0.46-2.2	0.975	0.93		0.37-2.3	0.876
**Time from last vaccine dose (more than 120 days as reference)**		0.68	0.37-1.2	0.208	–		–	–
**SARS-CoV-2 infection prior to 12^th^-months sample**		**0.15**	**0.07-0.31**	**<0.001**	**0.14**		**0.07-0.30**	**<0.001**
Immunosuppressive treatment								
Antimetabolite (None as reference)								
- Mycophenolate - Azathioprine	0.68 0.72		0.30-1.5 0.24-2.2	0.329 0.570	- -	- -		- -
**Corticosteroids**	1.9		0.98-3.6	0.057	1.5	0.66-3.6		0.319
CNI or mTOR (CNI as reference)								
mTOR	1.6		0.60-4.4	0.344	–	–		–
Comorbidities								
**Cardiovascular disease**	1.3		0.73-2.4	0.341	–	–		–
**Diabetes mellitus**	0.98		0.49-2.0	0.958	–	–		–

**Adjusted for age, sex, transplantation type, time from transplantation, number of vaccine doses, prior infection and corticosteroid treatment. P-values for the bold values were <0.05 (statistically significant).

### 3.8 Risk factors for IFN-γ non response

To determine risk factors of T-cell non-response 12 months after first vaccine dose logistic regression analyses were performed. In the uni- and multivariable logistic regression models, only diabetes mellitus (adjusted OR 2.4 [95% CI, 1.02-5.5], P=0.044) was associated with a IFN-γ non response (**Table 4**).

**Table 4:** Risk factors for T-cell non response.

	Unadjusted model			Adjusted model**			
	OR	95% CI	P-value	OR		95% CI	P-value
**Age per ten years**	1.1	0.84-1.5	0.490	1.1		0.82-1.5	0.531
**Female sex**	1.4	0.75-2.6	0.295	1.3		0.68-2.5	0.425
Transplantation type (Liver Tx as reference)							
- Kidney - Lung	0.75 1.5	0.38-1.5 0.61-3.5	0.412 0.395	0.72 1.4		0.35-1.5 0.55-3.4	0.359 0.509
**Time from transplantation to first vaccine dose**	0.97	0.93-1.02	0.195	0.98		0.93-1.02	0.298
Number of vaccines before the infection (Three doses as reference)							
- Four	0.82	0.32-2.1	0.672	0.99		0.37-2.7	0.988
**Time from last vaccine dose (more than 120 days as reference)**	1.3	0.64-2.5	0.497	–		–	–
**SARS-CoV-2 infection prior to 12^th^ month-sample**	0.84	0.42-1.7	0.618	0.86		0.41-1.8	0.673
Immunosuppressive treatment							
Antimetabolite (None as reference)							
- Mycophenolate - Azathioprine	1.02 1.2	0.45-2.3 0.37-3.1	0.955 0.804	- -	- -		- -
**Corticosteroids**	1.7	0.79-1.7	0.120	–	–		–
**CNI or mTOR** (CNI as reference)	0.69	0.25-1.9	0.465	–	–		–
mTOR							
Comorbidities							
**Cardiovascular disease**	1.2	0.65-2.4	0.523	–	–		–
**Diabetes mellitus**	**2.4**	**1.05-5.3**	**0.038**	**2.4**	**1.02-5.5**		**0.044**

**Adjusted for age, sex, transplant type, time from transplantation, number of vaccine doses, prior infection and diabetes mellitus. P-values for the bold values were <0.05 (statistically significant).

## 4 Discussion

In this prospective study, we included a large cohort of SOT recipients and matched controls and followed them 12 months after the first BNT162b2 vaccine dose. All participants received at least three doses of BNT162b2. During the entire follow-up, anti-RBD IgG GMCs were lower in SOT recipients than in controls. However, anti-RBD IgG in participants with prior SARS-CoV-2 infection was higher than in uninfected participants, and anti-RBD IgG GMC in SOT recipients with prior SARS-CoV-2 infection was comparable to that of controls with previous infection. For anti-RBD IgA, differences in response between SOT recipients and controls were less pronounced, although the anti-RBD IgA GMC was lower in SOT recipients than in controls at 12^th^-month. SOT recipients and controls with previous SARS-CoV-2 infection had comparable anti-RBD IgA GMCs at 12^th^-month. Importantly, inferior T-cell response was found in SOT recipients at 12^th^-month, even in participants with previous SARS-CoV-2 infection. Risk factors associated with immune non response varied. Previous SARS-CoV-2 infection was associated with lower risk of immune non responses, while the most important risk factors for immune non responses were age, female sex, being kidney or lung transplant recipient, diabetes mellitus, mycophenolate treatment, and time from transplantation to the first vaccine dose.

Anti-RBD IgG response was generally lower in SOT recipients than in controls, which aligns with current knowledge about antibody response to SARS-CoV-2 vaccination in SOT recipients (12, 46). Anti-SARS-CoV-2 antibodies increase after the third vaccine dose in SOT recipients (12, 47), but due to decay in short-lived plasma cells, the serum antibody concentrations usually decline in some months (48). Intriguingly, we found that the anti-RBD IgG response in SOT recipients with previous SARS-CoV-2 infection was higher than in SOT recipients without infection at 12^th^-month after the first vaccine dose. Importantly in SOT recipients with previous SARS-CoV-2 infection, anti-RBD IgG responses were comparable to that in controls. In line with our finding, previous studies showed that SARS-CoV-2 infection before or after vaccination boosts the humoral immune response and elicits broader immunity than vaccination alone (13, 29, 30, 49). In our study, only 15% of SOT recipients received the fourth vaccine doses, and the proportions of participants with previous SARS-CoV-2 infection were comparable between SOT recipients and controls. Thus, higher exposure to SARS-CoV-2 antigens in SOT recipients is unlikely to be a major confounder.

At baseline, there was a difference in anti-RBD IgG GMCs between SOT recipients and controls without previous infections. There may be several reasons for this finding. First, there is a difference in the timing of the first vaccine dose between the two groups. Second, it is possible that some participants had SARS-CoV-2 asymptomatic infections or had the infection in the spring of 2020 when there was limited testing capacity for SARS-CoV-2 in Denmark. It has been shown that the half-life of N-antibodies is shorter than the half-life of S-antibodies (50, 51). Therefore, it is possible that few participants tested negative in the N-antibody and did not have a positive SARS-CoV-2 PCR test, although they were infected during the spring of 2020.

The neutralizing capacity of antibodies is an important factor in preventing symptomatic SARS-CoV-2 infection and severe COVID-19 disease (52–54). Although the neutralization index at 12^th^-month was close to 99%, it was significantly lower in SOT recipients than in controls. Combined infection- and vaccination-derived immunity has been shown to induce high-quality antibodies and neutralize SARS-CoV-2 strains (54–56). Importantly, the neutralizing indices were comparable between SOT recipients and controls with the previous SARS-CoV-2 infection but not in participants without previous infection, highlighting the role of combined infection and vaccination (hybrid) immunity.

IgA has an important role in mucosal immunity against SARS-CoV-2 infection, and there is a positive association between serum and mucosal IgA concentrations (19, 57, 58). We measured serum anti-RBD IgA; 12 months after the first vaccine dose, and the anti-RBD IgA GMCs were lower in SOT recipients than in controls, albeit the differences were not as pronounced as for anti-RBD IgG. The anti-RBD IgA GMCs were higher in participants with previous SARS-CoV-2 infection than those without previous infection, again highlighting the impact of hybrid immunity. Considering the role of IgA antibodies in local protection, participants with hybrid immunity may have a lower risk of new SARS-CoV-2 infection and person-to-person transmission of SARS-CoV-2 (19, 58).

As part of adaptive immunity, T-cells have an important role in the clearance of SARS-CoV-2, preventing infection even without seroconversion, helping to mount a robust memory, and recognizing viral variants (24, 59, 60). It has been reported that on a median of five months after the second mRNA vaccine dose, only 13% of SOT recipients had T-cell response (28). In a previous paper, we reported that 13% of SOT recipients and 59% of controls receiving two vaccine doses were T-cell responders six months after the first vaccine dose (34). However, in the present study, with 12 months of follow-up, we showed that 46% and 69% of SOT recipients and controls were T-cell responders respectively. It should be noted that many of the participants had SARS-CoV-2 infection after the second vaccine dose and received one or two extra vaccine doses. SOT recipients with previous SARS-CoV-2 infection had higher T-cell responses than uninfected SOT recipients, although responses remained lower than in controls. The lower T-cell response in SOT recipients could be partly explained by the fact that most immunosuppressants inhibit T-cells (61). A delayed and inadequate T-cell response to SARS-CoV-2 infection has been reported to be associated with early inflammation and poor clinical outcome (24). Therefore, strategies to improve T-cell response in SOT recipients are warranted.

The adaptive immune response to vaccines is a complex process which is resulted from the interactions between antigens, antigen-presenting cells, cytokines, B cells, and T cells (48). A factor can affect the whole process or just a part, resulting in humoral or T-cell non responses. We identified factors associated with humoral or T-cell non responses. We showed that the female sex was associated with a lower likelihood of anti-RBD IgG response but not associated with anti-RBD IgA and T-cell responses. The association between sex and antibody response to SARS-CoV-2 vaccination has been reported in a number of previous publications, and data is conflicting (12, 62–65). In a recent meta-analysis including 26 studies on SOT recipients, the male gender was associated with a higher likelihood of antibody response after two doses of SARS-CoV-2 mRNA vaccines (12). Although, another recent meta-analysis by Zong et al. including 29 studies found that gender was not associated with antibody response after two doses of SARS-CoV-2 mRNA vaccines (64). In the study by Meunier et al., two-thirds of SOT recipients received more than three vaccine doses, and male gender was associated with a higher likelihood of positive (>260 BAU/ml) antibody response (63). However, females are known to have more robust immune responses to antigens due to the effects of sex hormones and X-linked genes (66, 67), and we cannot exclude that our finding is due to confounding factors that we have not accounted for. Kidney or lung transplant recipients were associated with the humoral non response but not with the T-cell non response, which corroborates well with previous reports (10, 18, 34, 68, 69). In our study, kidney transplant recipients had the highest proportion of cardiovascular diseases, and lung transplant recipients more commonly received CNI/mTOR inhibitors and corticosteroids, which could partly explain the differences in the immune response. In line with previous studies, mycophenolate treatment was associated with a higher chance of anti-RBD IgG non response (63, 64, 70). Mycophenolate potently inhibits antibody production, which can explain the higher chance of low anti-RBD IgG after vaccination (71). Age, time from transplantation to the first vaccine dose, and previous SARS-CoV-2 infection were factors that only affected the likelihood of anti-RBD IgA response. Finally, diabetes was the only factor associated with a decreased T-cell response. Hyperglycemia, glycemic variability, and some antidiabetic medications can affect T-cell function, increase the number of senescent T-cells, reduce T-cell lysis, and impair T-cell migration (72). Information about T cell response to vaccines other than SARS-CoV-2 in patients with diabetes is scarce. It has been reported that T cell response to primary protein antigens such as diphtheria toxoid is reduced in patients with diabetes (73).

Long-term follow-up of a large cohort of SOT recipients, inclusion of matched controls, and providing data on both anti-RBD IgG, anti-RBD IgA, neutralizing index, and T-cell response within 12 months after the first vaccine dose were the strengths of this study. Furthermore, results about anti-RBD IgA and T cell response in participants who had previous SARS-CoV-2 infection have not previously been reported in the literature. However, our study also had limitations; we could not match our controls on the number of vaccine doses and infections. Moreover, we could not investigate the kinetics of T-cell response.

In conclusion, the humoral and T-cell responses to at least three BNT162b2 vaccine doses were inferior in SOT recipients when compared to controls 12 months after the first vaccine dose. However, the antibody response was comparable in SOT recipients and controls with previous SARS-CoV-2 infection, while the T-cell response remained lower even in SOT recipients with previous SARS-CoV-2 infection. Factors associated with immune non response include transplant type, diabetes, and treatment with mycophenolate. Thus, increased attention toward maintaining high vaccination adherence in SOT recipients and toward the risk of breakthrough infections in this group is warranted.

## Data availability statement

The raw data supporting the conclusions of this article will be made available by the authors, without undue reservation.

## Ethics statement

The studies involving human participants were reviewed and approved by Regional Scientific Ethics Committee of the Capital Region of Denmark (H-20079890). The patients/participants provided their written informed consent to participate in this study.

## Author contributions

OR, SH, MP, KI, HB, SS, AR, and SN designed the study. Data was collected by SH, LDH, LP-A, DM, JM, AH, CH, MP-H, RH, KF, RF-S, LMH, ES, SO, and ZH. OR and SH analyzed the data. JA designed the statistical models. The first draft was written by OR, SH, and SN. All authors contributed to the article and approved the submitted version.
