# Supplementary material for: Humoral and T-cell response 12 months after the first BNT162b2 vaccination in solid organ transplant recipients and controls: Kinetics, associated factors, and role of SARS-CoV-2 infection
Source: Front Immunol. 2023 Jan 13;13:1075423. doi: 10.3389/fimmu.2022.1075423 (PMC9880190; doi:10.3389/fimmu.2022.1075423)
Supplement: Supplementary file 1 [file Table_1.docx]

Supplementary Material

1. Characteristics of participants who were matched on age and sex for IgA analyses

|  | SOT recipients  N= 204 | Controls  N = 204 | P-value |
| --- | --- | --- | --- |
| **Age, median (IQR)** | 58 (51-64) | 57(50-64) | 0.467 |
| **Sex (male), n (%)** | 118 (58%) | 120 (59%) | 0.920 |
| **Median time from first vaccine dose to sample time, days (IQR)** |  |  |  |
| - **Baseline** | -19 (-35-1) | 0 (0-0) | <0.001 |
| - **Three weeks** | 20 (19-22) | 23 (21-27) | <0.001 |
| - **Two months** | 59 (53-64) | 62 (57-65) | 0.002 |
| - **Six months** | 173.5 (157-201) | 165 (155-188.5) | 0.369 |
| - **Twelve months** | 366.5 (339.8-379.2) | 379 (372-386) | <0.001 |

1. Characteristics of participants who were matched on age and sex for IgG analyses

|  | SOT recipients  N=181 | Controls  N=181 | P-value |
| --- | --- | --- | --- |
| **Age, median (IQR)** | 58 (51-65) | 57(51-64) | 0.508 |
| **Sex (male), n (%)** | 102 (56%) | 104 (58) | 0.915 |
| **Median time from first vaccine dose to sample time, days (IQR)** |  |  |  |
| - **Baseline** | -20 (-35-0) | 0 (0-0) | <0.001 |
| - **Three weeks** | 20 (19-22) | 23 (21-27) | <0.001 |
| - **Two months** | 59 (52.5-64) | 62 (57-65) | 0.001 |
| - **Six months** | 172.5 (157-189) | 165 (155-189) | 0.614 |
| - **Twelve months** | 366 (339-379) | 379 (372-385) | <0.001 |

1. Characteristics of participants who were matched on age and sex for T cell analyses

|  | SOT recipients  N= 169 | Controls  N = 169 | P-value |
| --- | --- | --- | --- |
| **Age, median (IQR)** | 57 (49-63) | 56 (49-62) | 0.265 |
| **Sex (male), n (%)** | 98 (58%) | 98 (58%) | 1.00 |
| **Median time from first vaccine dose to sample time, days (IQR)** |  |  |  |
| **- Twelve months** | 365 (338-377) | 379 (372-386) | <0.001 |

**Sensitivity Analyses**

**Modelled anti-RBD IgG geometric mean concentration [AU/ml] with 95% confidence interval**

| Sample-time | SOT | Control | p-value |
| --- | --- | --- | --- |
| Baseline | 1.3 (1.2-1.6) | 4.3 (3.2-6.0) | 0.007 |
| 12-months | 1751 (1072-2696) | 15012 (1206-18484) | <0.001 |

Previous infection

| Sample-time | SOT | Control |  |
| --- | --- | --- | --- |
| Baseline | 115 (17-504) | 143 (82-228) | 0.508 |
| 12-months | 47407 (27300-82119) | 36565 (28143-48050) | 0.446 |

No previous infection

| Sample-time | SOT | Control |  |
| --- | --- | --- | --- |
| Baseline | 1.2 (1.1-1.3) | 3.2 (2.4-4.4) | 0.036 |
| 12-months | 939 (553-1478) | 11310 (9088-13972) | <0.001 |

#################################################################################

**Modelled anti-RBD IgA geometric mean concentration [AU/ml] with 95% confidence interval**

| Sample-time | SOT | Control |  |
| --- | --- | --- | --- |
| Baseline | 1.1 (1.1-1.3) | 1.2 (1.1-1.3) | 0.079 |
| 12-months | 15 (11-23) | 30 (21-43) | 0.003 |

Previous infection

| Sample-time | SOT | Control |  |
| --- | --- | --- | --- |
| Baseline | 2.5 (1.5-5.1) | 2.7 (1.6-5.7) | 0.594 |
| 12-months | 343 (199-565) | 360 (243-517) | 0.404 |

No previous infection

| Sample-time | SOT | Control |  |
| --- | --- | --- | --- |
| Baseline | 1.1 (1.0-1.1) | 1.1 (1.0-1.2) | 0.115 |
| 12-months | 5.4 (3.9-7.7) | 11 (7.8-17) | 0.009 |

**#################################################################################**

**Spike-specific IFN-g [mIU/mL] with IQR**

| Sample-time | SOT | Control | p-value |
| --- | --- | --- | --- |
| 12-months | 103.7 (0-794.3) | 1138.2 (488.1-2836.3) | <0.001 |

Previous infection

| Sample-time | SOT | Control |  |
| --- | --- | --- | --- |
| 12-months | 712 (79-3966) | 2657 (734-5285) | 0.031 |

No previous infection

| Sample-time | SOT | Control |  |
| --- | --- | --- | --- |
| 12-months | 46 (0-442) | 1020 (470-2030) | <0.001 |
